# Retraction: Baicalein Reduces the Invasion of Glioma Cells via Reducing the Activity of p38 Signaling Pathway

**DOI:** 10.1371/journal.pone.0160194

**Published:** 2016-07-25

**Authors:** The PLOS ONE Editors

After the publication of the article, a reader raised concerns about several figures in this article:

- In Figure 5A, the p-p38-MAPK panel appears to be the same as the TIMP-1 panel in Figure 6C, when flipped horizontally.
- In Figure 4B, the MMP-2 panel appears to be the same as the TIMP-2 panel in Figure 6C, when flipped horizontally.

These concerns were brought to the attention of the corresponding author, Zongfang Li, who stated that the submission was handled by the first author Zhenni Zhang without the knowledge of the other listed authors. An institutional investigation conducted by the Second Affiliated Hospital School of Medicine, Xi’an Jiaotong University indicated that Zhenni Zhang submitted the article without the knowledge of the listed co-authors and that some of the work was completed by an external company. Ms. Zhang takes full responsibility for these actions. Upon follow up, the editors have not had access to the data underlying the results reported in the article.

In light of the concerns identified and the lack of original data from the study, the *PLOS ONE* Editors have serious concerns regarding the integrity of the work and as a result retract this publication.
